# A metagenomic analysis of the gut microbiota in a mouse model of fish allergy

**DOI:** 10.3389/fmicb.2026.1886359

**Published:** 2026-09-01

**Authors:** Ana G. Abril, Javier Freire, Susana Magadán, Tomás G. Villa, Manuel Pazos, Mónica Carrera

**Affiliations:** 1 Department of Functional Biology and Health Science, Universidade de Vigo, Vigo, Spain; 2 Department of Food Technology, Spanish National Research Council (CSIC), Marine Research Institute (IIM-CSIC), Vigo, Spain; 3 Immunology Group, Center for Research in Nanomaterials and Biomedicine (CINBIO), University of Vigo, Pontevedra, Spain; 4 Immunology Research Group, Galicia Sur Health Research Institute (IIS Galicia Sur), SERGAS-UVIGO, Pontevedra, Spain; 5 Department of Microbiology and Parasitology, Faculty of Pharmacy, University of Santiago de Compostela, Santiago de Compostela, Spain

**Keywords:** beta-parvalbumin, fish allergy, food allergy, genomics, gut microbiota

## Abstract

**Background:**

Fish are among the most frequent causes of immunoglobulin E (IgE)-mediated food allergies (Type I). Currently, there is no known cure for fish allergy and individuals who are sensitized have to practice strict, lifelong avoidance of fish products in their diets. The relationship between gut microbiome and food allergies is currently a major topic of discussion; these pathologies involve the development of dysbiosis, which is a microbial imbalance resulting from immune-related mechanisms. Recent studies have provided evidence that individuals suffering from food allergies, display an intestinal microbiota with a different microbial composition compared to healthy subjects.

**Objectives and methods:**

In this work, we have described for the first time the differences in microbiome composition in a mouse model of fish allergy with previous sensitization to the main allergen, beta-Parvalbumin (*β*-PRVB), compared to mouse individuals without allergic response.

**Results:**

The metagenomic analysis has shown differences in taxonomic composition between the treatments. Regarding phyla, an increase in the relative abundance of *Patescibacteria,* specifically *Saccharimonas* genus and *Candidatus_Saccharimonas* group, were observed in the allergic animals (Prvb_Alum group) when compared to the other groups. In contrast, the relative abundance of the RF39 group (*Bacilli*), *Atopobiaceae* family, *Erysipelotrichaceae*, and the *Coriobacteriaceae*_UCG-002 group, was higher in the animals that did not develop an allergic response, despite being exposed to the allergen (Prvb group). Furthermore, an increase in the relative abundance of *Lachnospiraceae* ASF356 group was observed in the control group compared to the other treatments. This family, has been reported to be inversely associated with the progression of intestinal inflammation and anaphylactic diseases. For the first time, the gut microbiota composition of individual mice with and without fish allergies is described in detail in this work. This study may shed light on the potential contribution of gut microbiota to the onset and avoidance of food allergies.

## 1 Introduction

Food allergies reflect a state of hypersensitivity induced by food allergens. According to the World Health Organization (WHO), food allergy is the fourth largest public health issue, affecting 6–8% of the global pediatric population and 2–4% of adults. To date, the only effective treatment is following an allergen-free diet. The World Allergy Organization (WAO) highlights the high prevalence of these diseases in the last 20 years (Prescott, 2013; Prescott et al., 2013). The prevalence of food allergy is also gradually increasing in developing countries, a trend that cannot be solely attributed to genetic factors, with emerging research suggesting that alterations in the gut microbiota may play a role.

It is well established that contemporary lifestyle changes have led to alterations in the composition of gut symbiotic microorganisms (Liang et al., 2023). The balance and diversification within microbial communities (eubiosis) are crucial for the immune and metabolic homeostasis of the host, as well as for inhibiting pathogen penetration. In contrast, the dysregulation of microbiota composition (dysbiosis) is attributed to the onset of numerous diseases including allergies (Abril et al., 2023). The connection between the gut microbiome and food allergies is a prominent area of research. Recent research shows that individuals with food allergies have an intestinal microbiota with a distinct microbial composition compared to that of healthy individuals (Lee et al., 2020).

Determining causation in human studies is challenging; however the use of murine models may provide key information to understand how the intestinal microbiota influences food allergy. Studies with antibiotic-treated mice suggest that microbiota may suppress allergic responses (Zhang et al., 2021). This is further supported by germ-free mice, which exhibit higher peanut-specific IgE and IgG levels compared to mice with a normal microbiota, making them more prone to peanut allergy (Stefka et al., 2014). To identify protective microbial components, germ-free mice were colonized with specific bacteria. Monocolonization with *Bacteroides uniformis* did not reduce peanut sensitization or anaphylaxis, but colonization with *Clostridia* species effectively mitigated peanut sensitization and allergy symptoms (Atarashi et al., 2013). Other studies have shown that species belonging to these two major bacterial groups, *Clostridiales* and *Bacteroidales*, can inhibit the development of food allergies in mice via the induction of a subset of ROR*γ*t+ T regulatory cells (Tregs), which suppress both GATA-3 (GATA binding protein 3) Tregs and IgE, while enhancing IgA production (Abdel-Gadir et al., 2019). Furthermore, the analysis of mice with a mutation in the IL-4 receptor *α*-chain (Il4raF709), which are prone to food allergies, revealed a distinct microbial signature in allergen-sensitized Il4raF709 mice compared to resistant wild-type mice. Transferring microbiota from sensitized susceptible mice to germ-free mice transmitted food allergy susceptibility, whereas microbiota from resistant mice did not (Noval Rivas et al., 2013; Mathias et al., 2011). Taken together, these findings emphasize the critical role of gut microbiota in modulating the risk of food allergy.

The steady increase in the prevalence of food allergies underscores the urgent need for better methods for their prevention, diagnosis, and treatment. The incorporation of high-throughput omics approaches has emerged as a novel strategy to advance the understanding of complex mechanisms in diseases. Furthermore, recent advances in the field of bioinformatics have enabled the integration, analysis, and interpretation of the obtained information, paving the way for the development of personalized medical treatments for food allergy (Dhondalay et al., 2018). The efficiency and improved affordability, due to advancements in omics data acquisition and the development of advanced analytical techniques to process the large volumes of data produced, have led to an increase in the use of these techniques to study a variety of human diseases, including asthma, allergic rhinitis, food allergies, and immunodeficiencies. The integrated use of omics, microbiology, immunology, and bioinformatics will establish new strategies to overcome current limitations in the study of food allergies and their relationship with the microbiome (Abril et al., 2023). Next-generation sequencing, which includes 16S rRNA and whole-genome sequencing, provides essential information about bacteria that cannot currently be cultured *in vitro* but can be readily identified from fecal samples (Mandal et al., 2015). Several metagenomic studies have been conducted on murine models and the human microbiome and its relationship with different food allergies (Abril et al., 2023). In this regard, it is noteworthy that no studies have yet examined the microbiome associated with numerous allergies, such as fish allergy. Fish is one of the most common triggers of immunoglobulin E (IgE)-mediated food allergies (Type I). At present, no cure exists for fish allergies, requiring those affected to maintain a strict, lifelong exclusion of fish products from their diets. Most of the studies conducted have focused on determining how changes in the composition of the microbiota in the murine model can affect susceptibility to developing allergies, or in other words, to sensitization (Abril et al., 2023).

In this study, changes in microbiota composition were evaluated in mice already sensitized to the PRVB fish allergen using a metagenomic approach, with the aim of identifying variations in the abundance of specific bacterial species, or groups of bacteria, associated with the allergic phenotype. The results provide a basis for future studies focused on identifying microbial targets for potential therapeutic interventions in food allergy.

## 2 Materials and methods

### 2.1 *β*-PRVB purification

*β*-PRVBs purification was prepared as previously described (Carrera et al., 2011). Briefly, 2.5 g of white fish muscle of *Merluccius merluccius* were homogenized in 5 mL of MilliQ water, for 1 min using an Ultra-Turrax device (IKA Werke, Staufen, Germany). The *β*-PRVBs were purified by taking advantage of their thermostability and heating the samples to 70 °C. After centrifugation at 16,000 g for 10 min (J221-M centrifuge, Beckman, Palo Alto, CA, USA), supernatants containing principally *β*-PRVBs were quantified by the BCA method (Sigma-Chemical Co., USA). The samples were analyzed in triplicate.

### 2.2 Animals and the sensitization protocol

Female BALB/c mice (5–6 weeks) were purchased from Janvier Lab (Saint-Berthevin Cedex, France) and housed in the animal facilities of the Bioexperimentation Service (Sbio) at CINBIO - University of Vigo under specific pathogen-free (SPF) conditions with free access to food and drinking water. All procedures were carried out in strict compliance with the European Union Directive 86/609/EEC and approved by the Ethics Committee for Animal Experiments of the University of Vigo, Spain, and validated by the Xunta de Galicia (Spain) with the registration code ES360570215601/21/INVMED.02/OUTROS04/SMM. The sample size was chosen to ensure the reproducibility of the experiments in accordance with the principles of replacement, reduction and refinement of animal ethical regulations.

**Figure 1:**
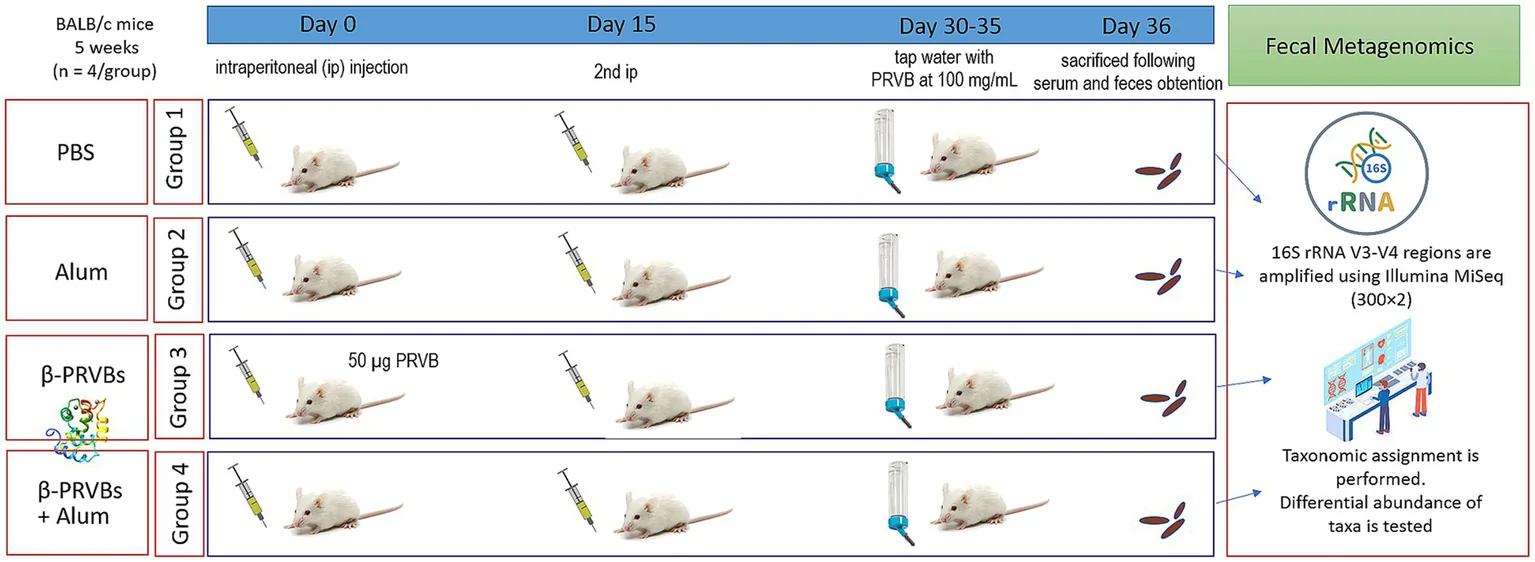
Scheme of the designed protocol in this study. Mice received two intraperitoneal (i.p) injections of: PBS (Group 1: Control) (*n* = 4); aluminum hydroxide—Al(OH)_3_ (Group 2: Alum) (*n* = 8); PRVB (Group 3: PRVB) (*n* = 8) and PRVB adsorbed to Al(OH)_3_ as adjuvant (Group 4: PRVB + Alum) (*n* = 8). All mice received an oral challenge with PRVB.

Five-week-old female BALB/c mice were maintained under SPF conditions. To be sensitized, the mice received two intraperitoneal (i.p) injections of 50 μg PRVB adsorbed to Al(OH)3 as adjuvant (Group 4: PRVB + Alum). One injection was administered on Day 0, and the second one 15 days after (i.e., on Day 15). Non-sensitized control groups were studied in parallel for comparison: mice intraperitoneal injection with saline (Group 1: Control), mice i.p. injected with aluminium hydroxide- Al(OH)3 (Group 2: Alum), mice i.p. injected with PRVB (Group 3: PRVB). All the mice received an oral challenge with PRVB. Specifically, at Day 30, and for 5 days (from 30 to 35), PRVB at 100 μg/mL was added in a bottle containing tap water (Figure 1). The day after, and before euthanasia by CO_2_ inhalation, feces and blood samples were collected. To collect feces, each mouse was placed in a single cage where the animal was allowed to defecate normally. The first 2–3 fecal pellets per animal were collected into empty Eppendorf tubes. The tubes containing the fecal pellets were stored at −80 °C until analysis (Abril et al., 2022).

Blood was obtained from the submandibular and/or retro-orbital plexus to obtain sera for humoral studies. The blood samples were kept at 4 °C for 2 h without anticoagulant. After the blood clot had completely formed, the samples were centrifuged at 1,000 × g for 10 min at 4 °C, the serum was collected, and a second centrifugation was performed at 1,200 × g for 2 min to completely remove all solid debris. The serum was then stored at −20 °C until analysis.

In this study, mouse groups and replicates were categorized into four experimental conditions: Control (PBS), Alum, Prvb, and Prvb_Alum. Each group was housed in specific cages, and individual replicates were denoted accordingly. The Control group consisted of four replicates (J1.R1–J1.R4), all housed in cage 1. To verify that microbiota composition changes are not induced by the environment, the procedure was performed twice (2 cages) in the following groups and fecal samples were collected from each individual animal. The Alum group included eight replicates (J2.R1–J2.R4 and J5.R1–J5.R4). The Prvb group also comprised eight replicates (J3.R1–J3.R4 and J6.R1–J6.R4), as did the Prvb_Alum group (J4.R1–J4.R4 and J7.R1–J7.R4).

### 2.3 PRVB specific antibodies determination

The *β*-PRVB-specific serum antibodies (IgE, IgG1, IgG2a and IgA) were measured after 0 and 28 days in mouse sera by ELISA. In brief, Maxisorp plates (Thermo Fisher Scientific Inc., Waltham, MA, USA) were coated with 100 μL *β*-PRVB (50 μg/mL) in a coating buffer, pH 9.6, overnight at 4 °C. The plates were washed and blocked with 5% skimmed milk (Merck, Darmstadt, Germany) in PBS for 2 h. After washing, 100 μL of the serum samples were diluted in a blocking solution at a dilution of 1:50 for IgE, 1:1,000 for IgG1, 1:200 for IgA and 1:100 for IgG2a. They were then incubated for 2 h. After washing, the secondary polyclonal antibodies bound to HRP were added at a dilution of 1:4,000 (goat anti-mouse IgG1-HRP, goat anti-mouse IgE-HRP, goat anti-mouse IgG2a-HRP, goat anti-mouse IgA-HRP, all of which originated from Southern Biotech, Birmingham, AL, USA). After washing, a color reaction was developed by adding TMB (Beckton Dickinson, Waltham, MA, USA) as a substrate for the HRP enzyme. The reaction was halted using 1 M H2SO4 (Merck, Darmstadt, Germany). The absorbance at 450 nm was measured using an EnVision 2104 microplate reader (Perkin Elmer, Waltham, MA, USA). The level of specific antibodies is expressed in arbitrary units.

### 2.4 Quantification of mMCP-1 in serum

Mucosal mast cell protease-1 (mMCP-1) was determined using a commercial mMCP-1 sandwich ELISA kit from Invitrogen (Thermo Fisher Scientific Inc., Waltham, MA, USA) according to the manufacturer’s instructions. The concentration of mMCP-1 was determined in the serum samples obtained after an oral challenge. Three technical replicates were performed for each sample.

### 2.5 *Ex vivo* stimulation of splenocytes with-parvalbumin

Single-cell splenocyte suspensions were prepared from sensitized animals after erythrocyte lysis. Splenocytes were seeded at a density of 2 × 10^6^ cells per well in 48-well flat-bottom plates containing 1 mL of RPMI-1640 medium supplemented with 10% fetal bovine serum and 1% penicillin/streptomycin. Cells were stimulated with *β*-PRVB at a final concentration of 20 μg/mL, while one well per sample was maintained without the allergen as a negative control. The cells were incubated at 37 °C in a humidified 5% CO_2_ atmosphere for 72 h. After the incubation period, culture supernatants were collected, centrifuged at 200 × g for 10 min to remove particulates, and stored at −80 °C until cytokine quantification.

### 2.6 Cytokine quantification

To obtain a cytokine profile, a Luminex multiplex bead-based immunoassay (ProcartaPlex Mouse Panel Invitrogen, Thermo Fisher Scientific) was also used to simultaneously quantify IL-1β, IL-4, IL-5, IL-6, IL-10, IL-13, and tumor necrosis factor-α (TNF-α). Briefly, 50 μL of supernatant was incubated in duplicate with cytokine-specific magnetic beads overnight at 4 °C. After washing, a biotinylated antibody mix was added for 30 min at room temperature with shaking (30 rpm), followed by streptavidin-phycoerythrin (PE) incubation under the same conditions. Beads were resuspended in reading buffer, and fluorescence was detected using a MAGPIX system (Luminex, Diasorin S.p.A., Saluggia, Italy). Cytokine concentrations were calculated from standard curves generated with a cytokine reference mix provided in the kit.

### 2.7 Meta-genomic analysis

#### 2.7.1 Library preparation and sequencing

DNA from fecal samples was isolated using MagMAX CORE Nucleic Acid Purification Kit 500 RXN (Thermo Fisher Scientific, CA, Austin, USA), following the protocol provided by the manufacturer. The extracted DNA were subsequently subjected to PCR amplification targeting the V3–V4 hypervariable regions of the 16S rRNA gene using region-specific primer pairs (V3-V4-Forward 5′-TCGTCGGCAGCGTCAGATGTGTATAAGAGACAGCCTACGGGNGGCWGCAG-3′, V3-V4-Reverse 5′GTCTCGTGGGCTCGGAGATGTGTATAAGAGACAGGACTACHVGGGTATCTAATCC-3′). Amplification was conducted using 25 PCR cycles. A negative control from the DNA extraction process, together with a positive Mock Community control*, was incorporated to monitor the quality of the procedure. Reactions were set up in a final volume of 10 μL, with primers at a concentration of 0.2 μM. The thermocycling program consisted of an initial denaturation at 95 °C for 3 min, followed by 25 cycles of 30 s at 95 °C, 30 s at 55 °C, and 30 s at 72 °C, ending with a final elongation phase of 5 min at 72 °C.

PCR amplicons were cleaned using AMPure XP beads (Beckman Coulter, Nyon, Switzerland) at a 0.9 × bead-to-sample ratio, following the manufacturer’s protocol. The primers described above included overhang sequences enabling the attachment of full-length Nextera barcoded adapters during a second PCR step, thereby generating sequencing-ready libraries with insert sizes of approximately 450 bp. In short, 5 μL of the cleaned first-round PCR product were used as a template for a secondary amplification employing Nextera XT v2 adaptor primers in a 30 μL reaction, using the same PCR master mix and cycling conditions as the initial PCR but reduced to eight cycles. Subsequently, 25 μL of the second PCR product were normalized and purified using the SequalPrep normalization kit (Invitrogen, Thermo Fisher Scientific, Waltham, MA, USA), according to the manufacturer’s instructions. Final libraries were eluted in 20 μL and pooled for sequencing. Sequencing was performed using Illumina MiSeq (2 × 300 bp) and v3 chemistry with a loading concentration of 10 pM.

#### 2.7.2 Amplicon sequences processing and analysis

Raw demultiplexed forward and reverse reads were analyzed using the workflows implemented in QIIME2 (version 2020.11), applying default settings unless otherwise specified (Bolyen et al., 2019; Quereda et al., 2020). DADA2 was employed to carry out quality filtering, denoising, pair-end merging and amplicon sequence variant calling (ASV, i.e., phylotypes) using the qiime dada2 denoise-paired method (Callahan et al., 2016). Q20 was employed as the quality threshold to define read sizes for trimming before merging (parameters: --p-trunc-len-f and --p-trunc-len-r). Reads were truncated at the position where the 75th percentile Phred score feel below Q20:292 bp for forward reads and 223 bp for reverse reads. After quality filtering steps, the average sample size was 74,095 reads (range: 30,213 to 120,257 reads) and a total of 3,526 phylotypes were identified. ASVs were aligned using the qiime alignment MAFFT algorithm (Katoh and Standley, 2013). The alignment served as the basis to create a tree and to calculate phylogenetic relations between ASVs using the qiime phylogeny fast tree approach (Price et al., 2010). ASV tables were subsampled without replacement to even sample sizes for diversity analysis using a qiime diversity core-metrics-phylogenetic pipeline. The sample with the smallest sample size (i.e., 30,210 reads) was discarded in order to take advantage of the sequencing depth of the dataset. Subsequently, subsampling to the next lowest sample size was used for each comparison. Unweighted and weighted Unifrac distances were calculated to compare community structure (Lozupone et al., 2011). The taxonomic assignment of ASVs was performed using a Bayesian Classifier trained with a Silva V4 database (i.e., 99% OTUs database) using the qiime feature-classifier classify-sklearn method (Pedregosa et al., 2011). Differential abundance of taxa was tested using a negative binomial linear model using the R MASS package version 7.3–58. Unifrac distance matrixes and ASV tables were used to calculate principal coordinates and construct ordination plots using the R software package version 4.2.0.^1^ The reported *p-*values correspond to raw (unadjusted) values. Given the exploratory nature of this study and the limited sample size, differential abundance analyses were primarily used to identify taxa potentially associated with the allergic phenotype. Therefore, results showing borderline statistical significance were interpreted cautiously and should be validated in larger cohorts. The significance of groups in community structure was tested using Permanova. BiodiversityR version 2.11-1 and vegan version 2.5–5 packages were used. ggplot2 version 2.2.1 was used for plotting. The raw reads were uploaded to the NCBI Sequence Read Archive (SRA) under the BioProject accession number PRJNA1280992.

Results were compared to identify a correlation between fish allergy developments and microbiome modifications and dysbiosis.

## 3 Results

In this study, the intestinal microbiota of *β*-PRVBs-sensitized animal models were examined, which may serve as a helpful guide for research on the intestinal flora of fish allergy. Most existing studies to date have focused on peanut, milk, and egg proteins (He et al., 2015; Gu et al., 2022), while few studies have examined how allergen sensitization affects the intestinal microbiota, particularly in animal models of fish allergy.

A marker-based approach using the 16S ribosomal RNA subunit gene (16S rRNA) was used to study the bacterial diversity of analyzed fecal samples. This approach allowed us to describe and quantify microbial alpha and beta diversity, and study taxonomic profiles from phylum to species levels in the presence of allergy reactions to *β*-PRVBs.

### 3.1 Allergic response in *β*-PRVB sensitized mice after allergen intake

**Figure 2:**
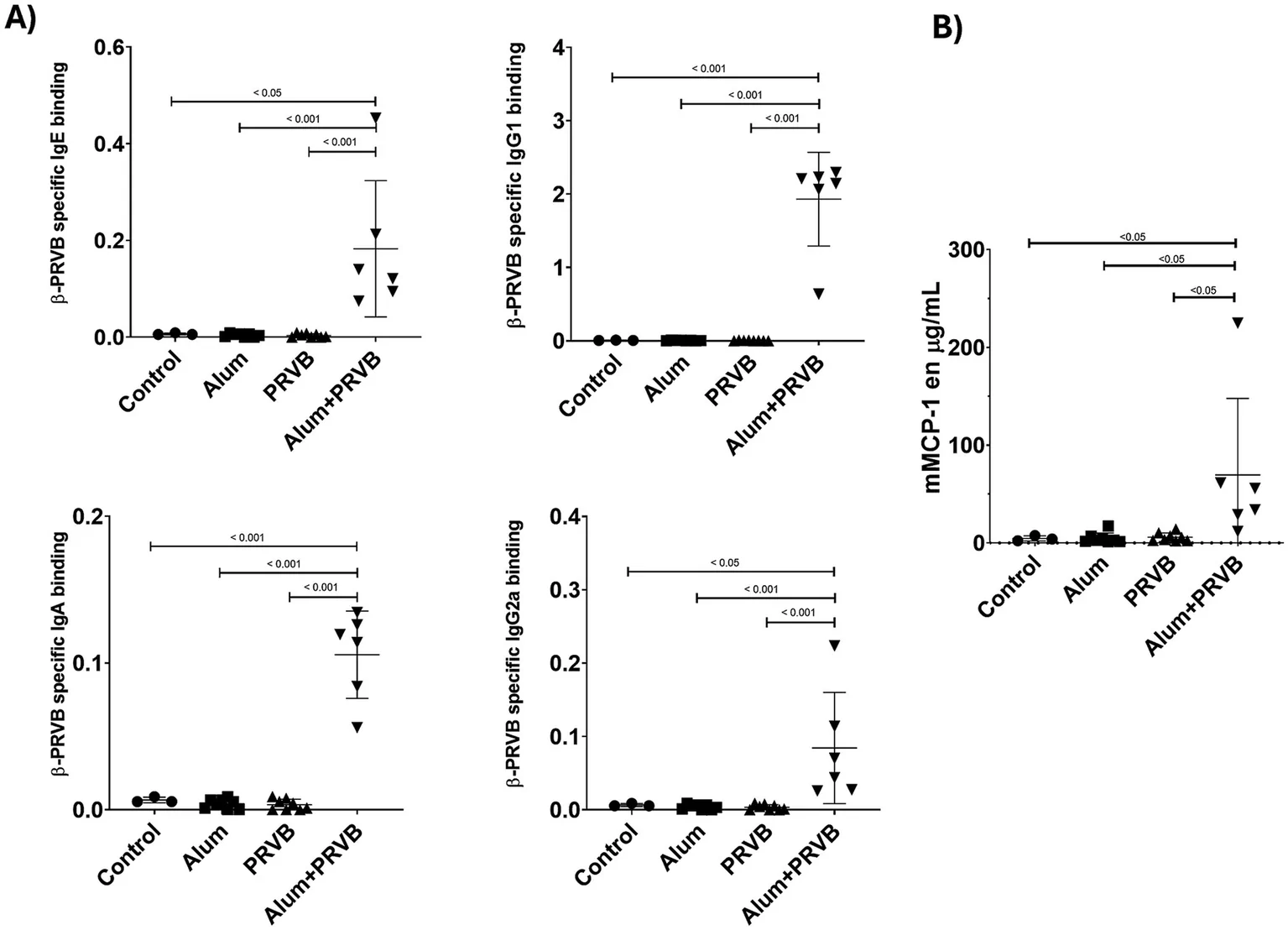
*β*-PRVB-specific immunoglobulins **(A)** and mouse mast cell protease 1 (mMCP-1) **(B)** levels in the serum of BALB/c mice subjected to a fish food allergy protocol. Serum dilutions, 1:50 for IgE, 1:1,000 for IgG1, 1:200 for IgA, and 1:100 for IgG2a were evaluated by indirect ELISA, and the results are represented as relative values to the most positive serum. PRVB-specific antibodies were undetectable in non-sensitized groups: PBS (*n* = 4), Allum (*n* = 8), and PRVB (*n* = 8). Concentration of mMCP-1 is expressed in μg/mL. The horizontal bar represents the mean concentration for each group with standard error. A one-way ANOVA test was performed, to determine statistical differences.

To investigate changes in the gut microbiota caused by fish food allergy, we established an allergy mouse model using the major fish allergen, *β*-PRVB (see Figure 1). After sensitization, mice were challenged for 5 days by the allergen administration in the drinking water to simulate the normal food intake. To evaluate the efficacy of this protocol we examined the humoral immune response following the administration of fish *β*-PRVB in water for 5 days. As expected, only the animals sensitized with two intraperitoneal injections of *β*-PRVB with the adjuvant Alum (PRVB/Alum) developed a clear allergen-specific IgG1 and IgE response, as well as allergen-specific IgA and IgG2a (Figure 2A),while, mice treated with PBS, Alum or *β*-PRVB lacked any specific humoral immune response.

Degranulation of mucosal mast cells resulting in acute elevation of blood levels of murine mucosal cell protease (mMCP)-1 after an allergen challenge has been identified as a biomarker of IgE-mediated systemic anaphylaxis in mice (Khodoun et al., 2011). Therefore, we determined mMCP-1 responses in mice based on oral allergen challenges. Although *β*-PRVB sensitized mice showed no obvious clinical signs of allergic response (data not shown), those mice with allergen-specific IgE in serum exhibited mMCP-1 responses upon undergoing the oral *β*-PRVB challenge (Figure 2B), while the non-effectively sensitized control mice did not show marked elevation of mMCP-1 levels following *β*-PRVB oral challenges (Figure 2B).

### 3.2 Cytokine profile of restimulated splenocytes

**Figure 3:**
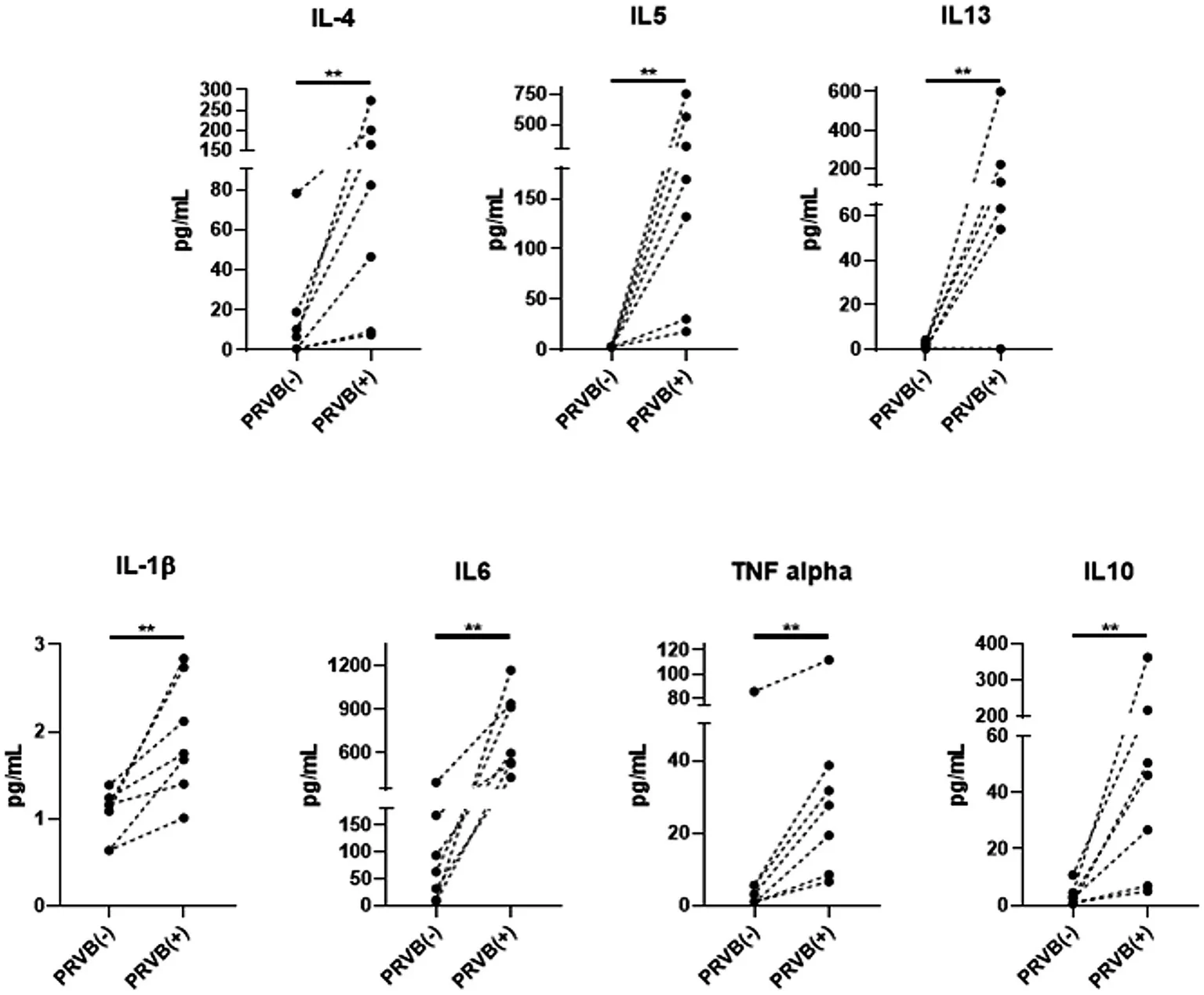
Cytokine levels in supernatant of cultured splenocyte obtained from sensitized mice after *β*-PRVB stimulation. Splenocytes from sensitized mice were cultured for 72 h with 20 μg/mL *β*-PRVB, and levels of relevant cytokines in the culture supernatant were measured by Luminex. Splenocytes from the same individuals were cultured in medium without *β*-PRVB as negative controls. Bars show mean ± SD, with each dot representing an individual value. The statistical analysis was performed using a one-tailed Wilcoxon matched-pairs signed rank test. Significance: ****p < 0.001; ***p < 0.005; **p < 0.01; *p < 0.05; n.s., non-significant.

A key feature of allergic response is the involvement of a Th2-like response. This response is characterized by the secretion of cytokines, primarily IL-4, IL-5 and IL-13, by T-helper cells upon recognition of allergen presented by antigen presenting cells (Tierney et al., 2026; Swain et al., 1990). We further characterized the cytokine profile produced by splenocytes from *β*-PRVB-sensitized animals using Luminex, which were cultured *in vitro* with or without *β*-PRVB (restimulation). We included Th2 associated cytokines (IL-4, IL-5 and IL-13), pro-inflammatory cytokines (IL-1β, IL-6 and TNF-α), and IL-10, a pleiotropic cytokine produced by many cell types including macrophages, epithelial cells, mast cells and regulatory T cells (Tregs), which had both pro- and anti-inflammatory effects. Overall, we observed an increase in all tested cytokines with inter-individual variability (Figure 3), suggesting a different proportion of cellular subsets or the variable nature of cytokine production following stimulation. Specifically, we observed a statistically significant increase in IL-4, IL-5, and IL-13 after incubation with *β*-PRVB, supporting the successful induction Th2-skewed and proinflammatory immune response.

### 3.3 Influence of the *β*-PRVBs challenge on bacterial diversity of sensitized mice

**Figure 4:**
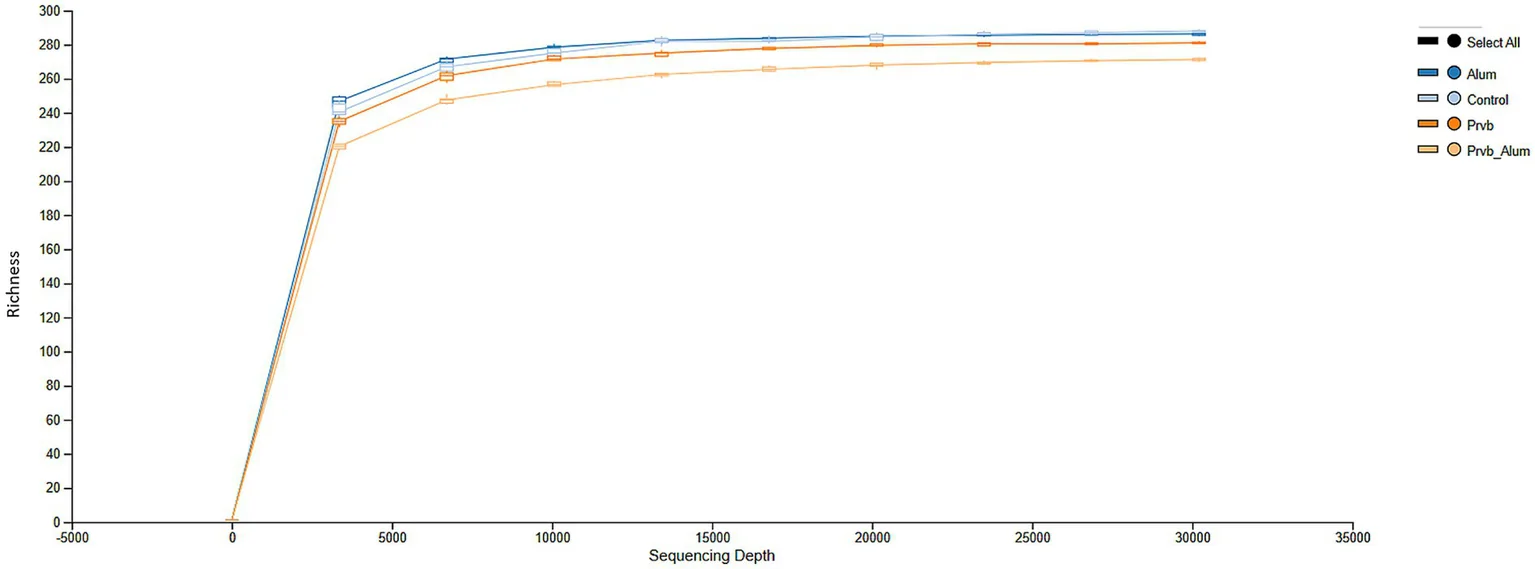
Rarefaction curves showing richness. The *X*-axis represents the sequencing depth (number of sequenced reads per sample), while the *Y*-axis displays the observed richness (number of distinct operational taxonomic units, OTUs). Each colored line represents a different group of samples. The plateauing of the curves indicates that sequencing depth was sufficient to capture the majority of the microbial diversity within the community.

Rarefaction curves indicated that the sequencing depth achieved, along with the selected subsampling threshold, was sufficient to capture the full extent of diversity within the microbial communities analyzed. A plateau was reached for the richness metric (Figure 4).

**Figure 5:**
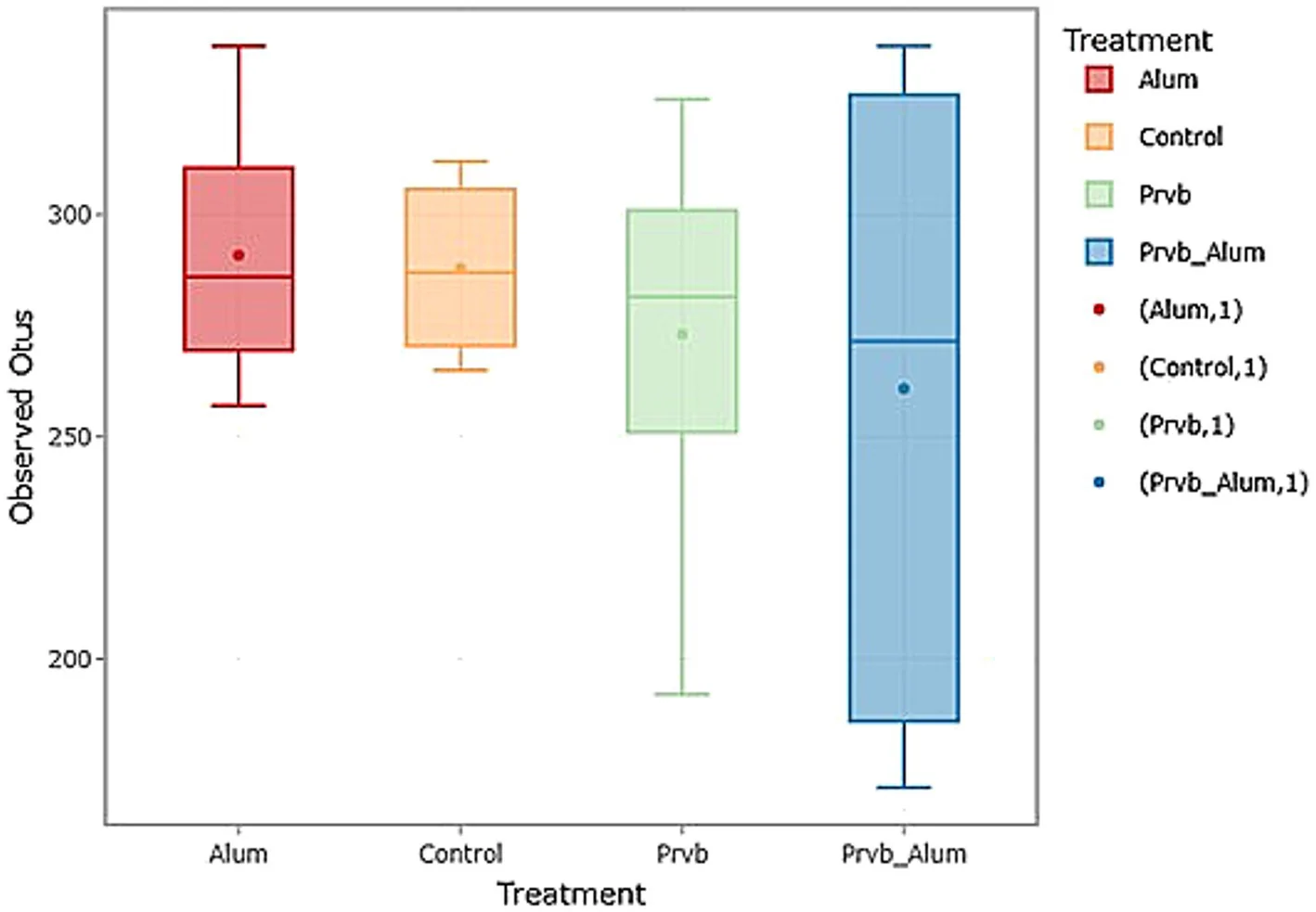
Richness (defined as the number of different species/phylotypes present in a community, here called Observed OTUs) of different group samples.

No significant differences were detected in richness or evenness among the sample groups (*p* < 0.05). Nonetheless, the variability in richness was markedly higher within the microbial communities in Prvb-Alum compared with the other group samples (Figure 5).

**Figure 6:**
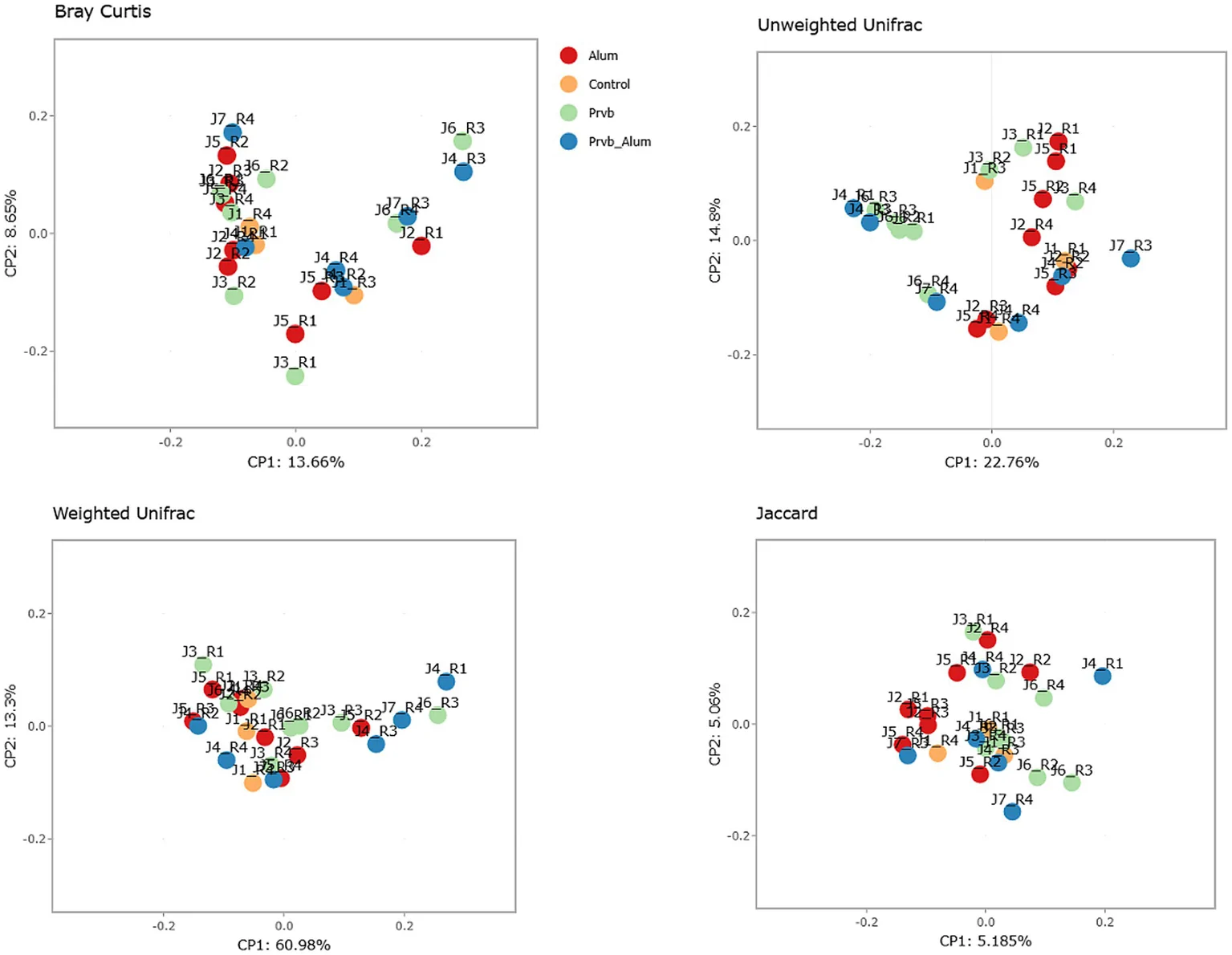
Beta diversity analysis. Principal coordinate analysis based on the Bray Curtis distance matrix, unweighted Unifrac distance matrix, weighted Unifrac distance matrix, and the Jaccard distance matrix. PBS (Group 1: Control) (*n* = 3); aluminum hydroxide—Al(OH)_3_ (Group 2: Alum) (*n* = 8); PRVB (Group 3: PRVB) (*n* = 8) and PRVB adsorbed to Al(OH)_3_ as adjuvant (Group 4: PRVB + Alum) (*n* = 6).

Analysis of community structure beta diversity, showed no differences between treatments (*p* < 0.05). The principal coordinate analysis based on the Bray Curtis distance matrix, unweighted Unifrac distance matrix, weighted Unifrac distance matrix, and Jaccard distance matrix is shown in Figure 6.

### 3.4 Influence of the *β*-PRVBs allergy on bacterial taxonomic profiles

The variable region targeted for amplicon sequencing in this study enabled the identification of bacterial taxa (Quereda et al., 2020). Bacteria were detected in 100% of samples. It was determined that the taxonomic composition of the samples should be examined by comparing the mean relative abundances of taxa across the respective groups with different treatments. The Excel data set in Supplementary Data 1 shows the relative abundance tables.

**Figure 7:**
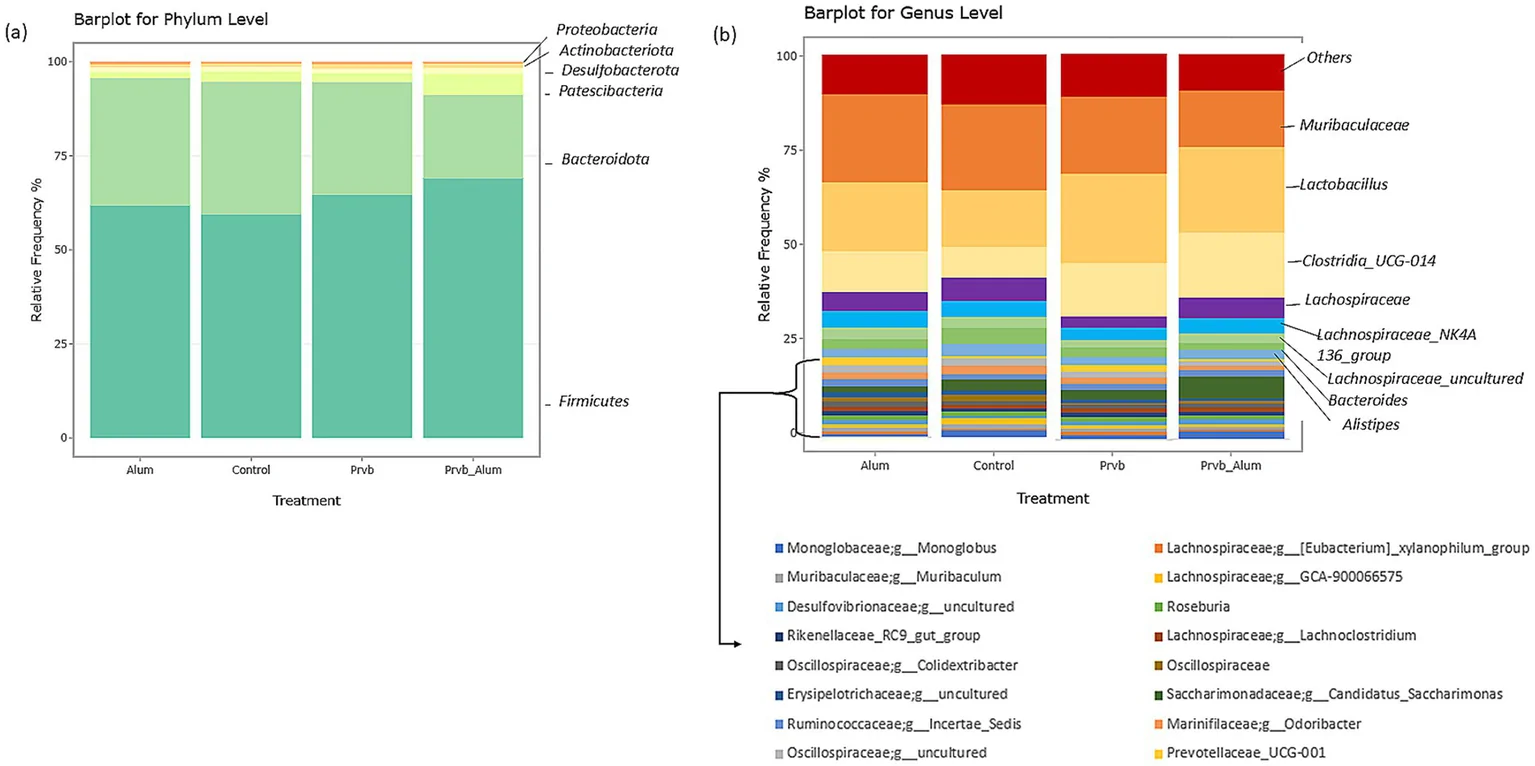
Relative abundance of taxa: **(A)** Relative abundance of phyla. **(B)** Relative abundance of genera. Only genus and families with a mean relative abundance >1.5% are shown.

Eight bacterial phyla were detected in the samples. The most abundant bacterial phyla were *Firmicutes*, and *Bacteroidota* (63.7 and 30.28%, respectively, computed as the mean relative abundance, Figure 7A and Supplementary Data 2), which accounted for more than 75%. The next most abundant phyla, in order of descending abundance, were *Patescibacteria Desulfobacterota, Actinobacteriota,* and *Proteobacteria*, which together with *Firmicutes*, and *Bacteroidota* spanned more than 99% of the bacterial taxa.

At the genus level or family level (when the genus or subsequent taxa could not be confidently assigned),nine genera and 13 families exhibited relative abundance >1% (Figure 7B and Supplementary Data 2). *Muribaculaceae, Lactobacillus, Clostridia, Lachnospiraceae*, NK4A136 group*, Bacteroides*, and *Alistipes* were the most abundant genera or families.

Differences in taxonomic composition (Supplementary Data 2 and Table 1) were observed between treatments. The observed differences in the community structure of the fish allergy murine model were consistent with previous observations (He et al., 2015; Gu et al., 2022; Vacca et al., 2020; Lee et al., 2023; Lee-Sarwar et al., 2023; Liu et al., 2023). Regarding phyla, differences were present between groups: *Firmicutes* (61.79, 59.44%, 64.61% and 69.01% of total bacteria in the Alum, control group, Prvb and Prvb- Alum, respectively); *Bacteroidota* (33.83%, 35.22%, 29.98% and 22.15% of total bacteria in the Alum, control group, Prvb and Prvb- Alum, respectively); and *Patescibacteria* (1.3%, 2.76%, 2.36% and 5.63% of total bacteria in the Alum, control group, Prvb and Prvb- Alum, respectively), where an increase in the relative abundance of *Patescibacteria* (*p-*value 0.029) was detectable in Prvb- Alum.

**Table 1:** Relative abundance comparison between taxa with relative abundance >0.05% (in minimum one treatment).

Taxa	Alum	Control	Prvb	Prvb_Alum
*Patescibacteria*	1.53125	2.76333	2.36625	5.63833
*Saccharimonadia*	1.53125	2.76333	2.36625	5.63833
*d__Bacteria;p__Actinobacteriota;c__Coriobacteriia;o__Coriobacteriales;f__Atopobiaceae*	0.00125	0	0.06375	0.00167
*d__Bacteria;p__Patescibacteria;c__Saccharimonadia;o__Saccharimonadales;f__Saccharimonadaceae*	1.53125	2.76333	2.36625	5.63833
*d__Bacteria;p__Actinobacteriota;c__Coriobacteriia;o__Coriobacteriales;f__Atopobiaceae;g__Coriobacteriaceae*_UCG-002	0.00125	0	0.06375	0.00167
*d__Bacteria;p__Firmicutes;c__Bacilli;o__Erysipelotrichales;f__Erysipelotrichaceae;g__Erysipelotrichaceae*	0.09125	0.16	0.295	0.09
*d__Bacteria;p__Firmicutes;c__Clostridia;o__Lachnospirales;f__Lachnospiraceae;g__*ASF356	0.1	0.28	0.0975	0.02833
*d__Bacteria;p__Firmicutes;c__Clostridia;o__Oscillospirales;f__Ruminococcaceae;g__*UBA1819	0.325	0.95667	0.85375	0.13667
*d__Bacteria;p__Patescibacteria;c__Saccharimonadia;o__Saccharimonadales;f__Saccharimonadaceae;g__Candidatus_Saccharimonas*	1.53125	2.76333	2.36625	5.63833
*d__Bacteria;p__Actinobacteriota;c__Coriobacteriia;o__Coriobacteriales;f__Atopobiaceae;g__Coriobacteriaceae_UCG-002;s__uncultured_bacterium*	0.00125	0	0.06375	0.00167
*d__Bacteria;p__Firmicutes;c__Bacilli;o__Erysipelotrichales;f__Erysipelotrichaceae;__;__*	0.01	0.09667	0.0925	0.29167
*d__Bacteria;p__Firmicutes;c__Clostridia;o__Lachnospirales;f__Lachnospiraceae;g__*ASF356;*s__uncultured_bacterium*	0.1	0.28	0.0975	0.02833
*d__Bacteria;p__Firmicutes;c__Clostridia;o__Lachnospirales;f__Lachnospiraceae;g__Roseburia;__*	0.245	0.01	0.12	0.005
*d__Bacteria;p__Firmicutes;c__Clostridia;o__Oscillospirales;f__Ruminococcaceae;g_*_UBA1819;*s__uncultured_bacterium*	0.325	0.95667	0.85375	0.13667

*p-*value < 0.05.

**Figure 8:**
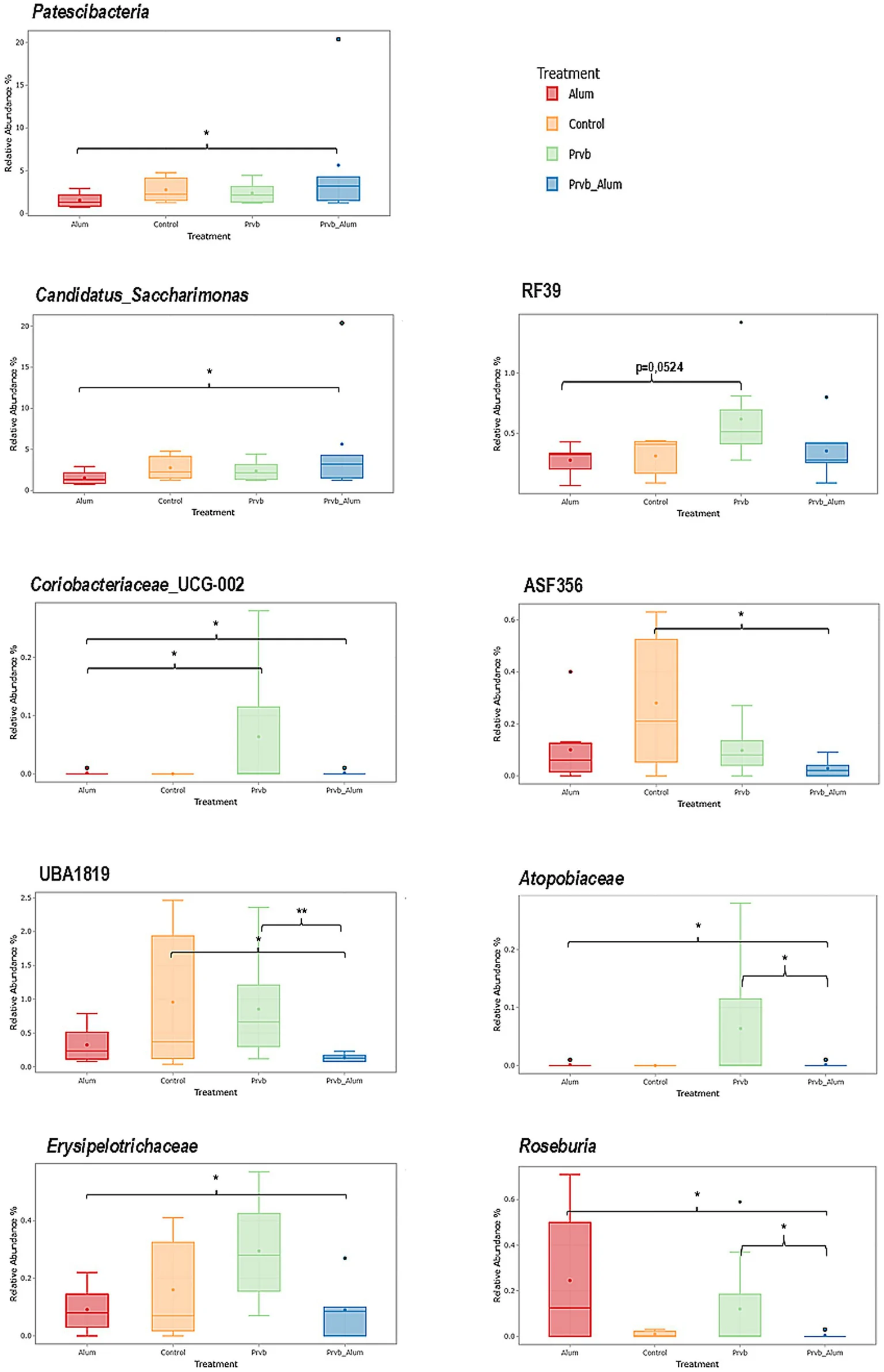
Taxa with significant differences in relative abundances between treatments. The ANOVA test was used to determine the statistical significance. *p < 0.05, **p < 0.01, ***p < 0.001, and ****p < 0.0001. Only taxa with a mean relative abundance > 0.05% are shown. The mean relative abundance is represented by a line inside the box.

Regarding genera, our results specifically showed an increase in the relative abundance of the *Saccharimonas* genus (*Patescibacteria*) and *Candidatus_Saccharimonas* group (previously known as the TM7 group (He et al., 2015; Hug et al., 2016) (*p* < 0.005, Supplementary Data 3) in feces samples from previously sensitized mice to PRVB (Prvb_Alum group) when compared to the other groups (Figure 8).

In contrast, the relative abundance of the RF39 group (*Bacilli*) (*p* < 0.052), *Atopobiaceae* family (*p* < 0.05), *Erysipelotrichaceae* (*p* < 0.05), and *Coriobacteriaceae*_UCG-002 group (*p* < 0.05) was higher in Prvb than in the other treatments. Furthermore, an increase in the relative abundance of the *Lachnospiraceae* ASF356 group was observed in control group compared to the other treatments.

Meanwhile, the relative abundance of the *Ruminococcaceae* UBA1819 group decreases in the treatments with Alum and Prvb_Alum compared to controls and the Prvb group. Finally, the relative abundance of *Roseburia* increased in the Alum treatment.

Table 1 displays the overview of the relative taxa abundance comparison between groups with relative abundance >0.05% and *p-*value < 0.05.

## 4 Discussion

After sensitization, using the *β*-PRVB, only the animals sensitized with two intraperitoneal injections of *β*-PRVB with the adjuvant Alum (PRVB/Alum) developed a clear allergen-specific response and exhibited mMCP-1 responses upon an oral *β*-PRVB challenge (Figure 2). These results suggest that sensitization with *β*-PRVB and Alum effectively primes the immune system for an acute IgE mediated allergic response upon subsequent exposure to the allergen by ingestion.

The relationship between gut microbiome and food allergies is currently a major topic of discussion and a microbial imbalance can result from immune-related mechanisms. In this sense, the taxonomic composition of the samples should be examined by comparing the mean relative abundances of taxa across the respective groups with different treatments.

Comparing the mean relative abundances of taxa, differences in taxonomic composition (Supplementary Data 2 and Table 1) were observed between treatments These data are consistent as a decrease in *Bacteroidota* and an increase in *Firmicutes* of total bacteria in food allergen sensitized mice was also found in recent studies; particularly in oral and gut microbiome from a 6-week-old BALB/c mice sensitised with ovalbumin (OVA) and Alum (Qiao et al., 2024). However, in another study in which BALB/c mice were sensitized with wheat gluten combined with Alum as an adjuvant, the abundance of *Firmicutes* in the wheat protein sensitized group was lower than that in the normal group, while the abundance of *Bacteroidetes* was higher; and *Lactobacillus* was the differential marker in the non-sensitized normal group (Liu et al., 2023). Yu et al. (2024) conducted a research into BALB/c mice in which epicutaneous sensitization with OVA significantly increased the relative abundance of *Bacteroidetes* while reducing the relative abundance of *Firmicutes* (Yu et al., 2024). Short chain fatty acids (SCFAs), although not evaluated here, may have a role in the results shown in the present paper. Indeed, the SCFAs and *Firmicutes* to *Bacteroides* ratio seems to be related in lean and obese human phenotypes, with higher *Bacteroides/Firmicutes* counts associated with lower fecal SCFAs (Fernandes et al., 2014). In our case, the putative results for *β*-PRVB sensitized mice would imply an increase in the intestinal SCFAs, consistent with the results reported by Trompette et al. (2014) where mice with decreased levels of SCFAs showed an increased response to allergic airway symptoms and hematopoiesis. As it has been reported, high values of SCFAs such as butyrate, alleviate asthma by inhibiting Tfh13-mediated IgE production (Yu et al., 2025). Also, butyrate alleviates PD-1/PD-L1 inhibitor-related cardiotoxicity via the PPARα-CYP4X1 axis in colonic macrophages (Chen et al., 2022).

Regarding genera, the increase of *Saccharimonas* (the genus name refers to its ability to use a variety of carbohydrates) in the Prvb_Alum treatment samples is notable as it has been previously detected in hospitals and many other anaerobic habitats, in periodontal diseases, dermatitis, cystic fibrosis or bowel diseases; consequently a pathogenic role is being assumed today (Wang et al., 2021). Moreover, an analysis of the salivary microbiome has revealed that *Candidatus_Saccharimonas* may act as a community driver for colorectal cancer in mice (Wang et al., 2021). Its epiphytic nature, characterized by the ability to grow on the surface of *Actinomyces* species and being an obligate epiphyte of *Actinomyces odontolyticus* (He et al., 2015), suggests a novel mechanism by which a small bacterium (cocci of 200–300 nm; comparable in size to a large virus with a genome of only 705 kb) could exert a pathogenic effect. In addition, *Saccharimonas* stimulated *Actinomyces* to produce TNF-α, indicating a potential role as an immune suppressor. Furthermore, it has been reported that *Saccharimonas* positively correlates with adaptive immunity markers, specifically CD4+ CD57+ T-cells (Xie et al., 2021).

A trend toward a higher relative abundance of the RF39 group (*Bacilli*) was observed in the Prvb group compared with the other treatments (*p* = 0.052), although this difference did not reach statistical significance. RF39 is a novel uncultured *Tenericutes* lineages of *Bacilli*, reported in the gut of humans and domestic animals (Xie et al., 2021). Wang et al. (2020) found that RF39 was prevalent in the gut microbiota, and that it might produce metabolites, such as acetate and hydrogen, that affect the amount of acetic acid, which is a short-chain fatty acid (SCFA) characteristic in the gut. However, the increase in relative abundance of the RF39 group in Prvb group, is not surprising, as a positive correlation between RF39 and increased inflammatory cytokines has been reported (Zhang et al., 2020). Furthermore, RF39 was associated with increased adiposity in rats fed a Western high-fat diet (Wang et al., 2017). In addition, RF39 was associated to significantly increased in the intestine of adult offspring of maternal mice that were fed a high-fat diet and is associated with steatohepatitis (Guzzardi et al., 2021). In other study, the relative abundance of RF39 was significantly increased in the intestines of rats with myocardial ischemia–reperfusion injury and significantly decreased after electroacupuncture treatment (Bai et al., 2021).

Likewise, the increase of the anaerobic *Atopobiaceae* family (*p* < 0.05) and *Coriobacteriaceae*_UCG-002 group (*p* < 0.05), contained within the *Coriobacteriales* order in the Prvb treatment group correlates with the fact that they have been described as part of the polymicrobial nature of periodontal diseases together with *Peptostreptococcus*, *Filifacter Megasphaera, Desulfobulbus, Campylobacter, Selenomonas, Deferribacteres, Dialister, Catonella, Tannerella, Streptococcus, and Eubacterium* (Kolenbrander et al., 2009). In the case of *Erysipelotrichaceae* (*p* < 0.05), a higher abundance was observed in the Prvb-treated samples compared with the controls. This may be relevant given that, although members of this family are often considered commensals, *Erysipelothrix rhusiopathiae* (Brooke and Riley, 1999) and *Bulleidia extructa* are recognized as primary human pathogens (Morgan and Goldstein, 2021).

Furthermore, an increase in the relative abundance of the *Lachnospiraceae* ASF356 group was observed in the control group compared to the other treatments. The *Lachnospiraceae* family belongs to the core of the gut microbiota. The abundance of this family has been reported to be inversely associated with the progression of intestinal inflammation and anaphylactic diseases (Guo et al., 2021). Furthermore, in a previous study*, Lachnospiraceae* was negatively correlated to the proportions of Th2 in the peripheral immune organ (Zhou et al., 2021). This, indeed, supports the idea of the beneficial effects of these bacteria (they are among the main producers of SCFA) that colonize the human intestinal lumen from birth and constantly increase during the host’s life (He et al., 2015). Six-week-old SPF BALB/c female mice were sensitized with peanut combined with Alum adjuvant via intraperitoneal injection and challenged twice. In accordance with the present study, peanut sensitized mice have shown a decrease in the presence of the *Lachnospiraceae* family (Gu et al., 2022). Moreover, BALB/c epicutaneous sensitization with OVA significantly decreases the abundance of *Lachnospiraceae* (Yu et al., 2024).

Meanwhile, the relative abundance of the *Ruminococcaceae* UBA1819 group decreased in the treatments with Alum and Prvb_Alum compared to the controls and the Prvb group. Regarding this, controversial results were obtained in different studies. The effect of an olive-derived antioxidant dietary fiber in relieving atopic dermatitis symptoms was evaluated and the genus abundance of *Ruminococcaceae UCG014*, *GCA900066575*, and *UBA1819* was restored by supplementation (Lee et al., 2023). On the other hand, it has been observed how an increased abundance of UBA1819 in the infant fecal microbiome has been strongly associated with the asthma phenotypes, and may indicate a higher risk of premature delivery, which could, in turn, elevate the risk of asthma in offspring (Lee et al., 2023).

Finally, *Roseburia* increased the relative abundance in the Alum treatment. The beneficial effects of SCFA in humans such as butyrate, are the presence of the main metabolite of *Roseburia* especially at a mildly acidic pH (Kettle et al., 2015).

These findings provide evidence of an association between *β*-PRVB-induced allergic sensitization and alterations in gut microbiota composition; however, several limitations should be considered. A limitation of the present study is the relatively small sample size, which may reduce the statistical power to detect subtle differences in microbial taxa, even though the allergic phenotype was consistently observed across animals and replicated in two independent cages. Larger cohorts, multiple-testing correction, and independent validation datasets will be required in future studies to validate these findings and to further characterize the contribution of low-abundance microbial populations to the observed effects.

Moreover, it remains unclear whether taxa changes contribute to disease progression, result from immune activation, or reflect broader physiological alterations associated with inflammation. Functional studies, such as fecal microbiota transplantation or targeted microbial modulation, would be necessary to determine causal relationships. Moreover, the use of a murine model, while advantageous for controlled sensitization and mechanistic investigation, imposes limitations on the direct translation of these findings to human food allergy. The composition and functional dynamics of the murine gut microbiota differ substantially from those of humans (Weingarden, 2026) and immune responses elicited under experimental conditions. Longitudinal studies and validation in human cohorts will be essential to determine whether the microbial signatures identified here could serve as biomarkers of susceptibility or targets for therapeutic intervention in food allergy. Additionally, future studies incorporating metagenomic, metabolomic, or transcriptomic approaches would help clarify the metabolic pathways and immunomodulatory mechanisms underlying the observed associations.

In summary, our results support a link between allergic sensitization to fish, an area that has not been previously explored, and gut microbiota composition. However, further investigation is required to elucidate the mechanistic basis and translational relevance of this interaction.

## 5 Conclusion

This is the first instance in which the fecal microbiota of a murine model for fish allergy has been analyzed. A high diversity of bacteria was observed, while no diversity of Archaea was detected. Differences in taxonomic composition were identified across different treatments. Individuals with fish allergy, who were those receiving Prvb_Alum treatment, exhibited a higher relative abundance of *Patescibacteria*, particularly the genus *Saccharimonas* and the group *Candidatus_Saccharimonas*, compared to other groups. Meanwhile, the Prvb treatment samples showed an increase in the RF39 group, *Atopobiaceae, Erysipelotrichaceae*, and the *Coriobacteriaceae*_UCG-002 group. In the control group, the *Lachnospiraceae* ASF356 group was more abundant than in other treatments. Additionally, the *Ruminococcaceae* UBA1819 group decreased in abundance in both Alum and Prvb_Alum treatments, while *Roseburia* showed a relatively higher abundance specifically in the Alum treatment.

An increase in the abundance of specific taxa has been noticed in groups of mice with heightened immune responses. The production of TNF-*α* in response to a microbial stimulus like *Saccharimonas* could have implications for inflammation regulation and various immune responses. *Actinomyces*, known for its role in oral microbiota and in some pathological contexts, may, by producing TNF-α in response to the presence of *Saccharimonas*, influence local or systemic inflammation. This interaction may be relevant in microbiome studies, inflammatory diseases, and the modulation of immune responses. In the control group, there was an increase in beneficial bacteria, particularly a higher relative abundance of the *Lachnospiraceae* ASF356 group when compared with other treatments. Members of the *Lachnospiraceae* family are a key component of the core gut microbiota and are thought to contribute to the maintenance of eubiosis as they have been reported to be inversely associated with the progression of intestinal inflammation and anaphylactic diseases. These bacteria may, therefore, hold therapeutic potential for food allergies associated with allergic dysbiosis by helping to restore intestinal eubiosis.

Many findings on the differences in gut microbiota composition between food allergen-sensitized BALB/c mice and control groups align with the results of the present study. These include a decrease in *Bacteroidota*, an increase in *Firmicutes*, and a reduced abundance of the *Lachnospiraceae* family in sensitized mice. However, variations in allergen type, dosage, and route of administration can lead to inconsistencies in the observed outcomes. To minimize these variations and improve reproducibility, future studies should standardize experimental protocols, including the choice of allergen, sensitization dose, administration route (e.g., oral, intraperitoneal), and duration of exposure. Additionally, including larger sample sizes may help better isolate the effects of specific variables on gut microbiota composition.

The present study is the first to describe specific differences in gut microbiota composition between individual mice with and without fish allergy. We believe this research can provide new insights into the role that gut microbiota may play in the development and prevention of food allergies.

## Data Availability

The datasets presented in this study can be found in online repositories. The names of the repository/repositories and accession number(s) can be found at: https://www.ncbi.nlm.nih.gov/, PRJNA1280992.

## Footnotes

1
http://www.R-project.org
